# *Foxp1* Regulates Cortical Radial Migration and Neuronal Morphogenesis in Developing Cerebral Cortex

**DOI:** 10.1371/journal.pone.0127671

**Published:** 2015-05-26

**Authors:** Xue Li, Jian Xiao, Henning Fröhlich, Xiaomeng Tu, Lianlian Li, Yue Xu, Huateng Cao, Jia Qu, Gudrun A. Rappold, Jie-Guang Chen

**Affiliations:** 1 Key Laboratory of Visual Science, National Ministry of Health, and School of Optometry and Ophthalmology, Wenzhou Medical University, Wenzhou, 325027, P. R. China; 2 Department of Human Molecular Genetics, University of Heidelberg, Im Neuenheimer Feld 366, 69120, Heidelberg, Germany; Osaka University Graduate School of Medicine, JAPAN

## Abstract

FOXP1 is a member of FOXP subfamily transcription factors. Mutations in *FOXP1* gene have been found in various development-related cognitive disorders. However, little is known about the etiology of these symptoms, and specifically the function of *FOXP1* in neuronal development. Here, we report that suppression of *Foxp1* expression in mouse cerebral cortex led to a neuronal migration defect, which was rescued by overexpression of *Foxp1*. Mice with *Foxp1* knockdown exhibited ectopic neurons in deep layers of the cortex postnatally. The neuronal differentiation of *Foxp1*-downregulated cells was normal. However, morphological analysis showed that the neurons with *Foxp1* deficiency had an inhibited axonal growth *in vitro* and a weakened transition from multipolar to bipolar *in vivo*. Moreover, we found that the expression of *Foxp1* modulated the dendritic maturation of neurons at a late postnatal date. Our results demonstrate critical roles of *Foxp1* in the radial migration and morphogenesis of cortical neurons during development. This study may shed light on the complex relationship between neuronal development and the related cognitive disorders.

## Introduction

Projection neurons in the mammalian cerebral cortex are generated from the progenitor cells in the ventricular and subventricular zone (VZ/SVZ), and then migrate radially toward the cortical plate (CP). Early-born neurons occupy deep cortical layers whereas later-generated neurons take more superficial layers, resulting in an “inside-out” pattern of cortical histogenesis [1]. Neurons arrive at proper positions and acquire their mature morphology in order to establish synaptic connections and form circuits. During the migration, newly-born projection neurons undergo an early morphological transition within the intermediate zone (IZ) [2,3]. The newly-generated neurons first become multipolar in the lower IZ and then change to a bipolar shape when axons start to grow out [4]. The bipolar neurons attach to radial glial cells and migrate outward from the IZ, reaching their destination in the CP [5,6]. This early polarization process in the IZ is critical for the migration and emergence of proper cortical lamination [7–10]. The migration and morphogenesis of cortical neurons are complex but precisely orchestrated processes, the impairments of which lead to severe brain malformations and neuropsychological disorders in humans, such as schizophrenia, epilepsy, autism, etc. [7,8,10]. The molecular and cellular mechanisms that regulate the development of cortical neurons, however, are not fully understood.

The FOXP1 protein is widely expressed in human and murine tissues and is involved in the regulation of development of multiple tissues and organs, namely heart, lung, esophagus and the immune system [11–15]. *Foxp1* is expressed in several regions of the central nervous system including the cerebral cortex, striatum and the spinal cord [13,16,17]. During development, *Foxp1* regulates motor neuron migration, midbrain dopamine neuron differentiation and striatum development [18–20]. All these studies provide compelling evidence that *Foxp1* may participate in critical phases of brain development. Consequently, malfunctions of *Foxp1* have been associated with a spectrum of development-related brain diseases. Point mutations, deletions and other disruptions of the *FOXP1* gene have been found in patients with global developmental delay, intellectual disability and autism spectrum disorders [21–27].

In this study, we have explored the role of *Foxp1* in mouse cortical development *in vivo* and found that inhibition of *Foxp1* expression in the cerebral cortex resulted in impairments of neuronal migration. *Foxp1* knockdown led to defects in the early polarization of neurons and the developmental maturation of neuronal processes. This study suggests that *Foxp1* may be a critical determinant regulating neuronal migration and morphogenesis during cortical development.

## Materials and Methods

### Animals and Ethics Statement

All animal experiments were approved by the Animal Care Committee of Wenzhou Medical University. The ICR mice used in this study were maintained on a 12-h light/dark cycle in a temperature-controlled room. The morning of the day when a vaginal plug was found was designated as embryonic day (E) 0.5. The day of birth was designated as postnatal day zero (P0). To relieve the pain associated with *in utero* electroporation, Ibuprofen (oral; 100 mg/kg in water) was provided to the pregnant mothers who had recovered on a heating pad from surgical anesthesia. Pregnant or postnatal mice were euthanized via lethal intraperitoneal injection of pentobarbital (50 mg/kg).

### Plasmids

Mouse *Foxp1* expression vector pCMV10-mFoxp1, the short hairpin interfering RNA constructs (pLKO.1_sh_ex13, pLKO.1_sh_ex15) and a scrambled control (pLKO.1_scramble shRNA), were purchased from Addgene and they were the gifts from Benjamin Blencowe and David Sabatini [28,29]. The targeting sequences of *Foxp1* shRNA-a and shRNA-b are 5′-GCTAACACTAAACGAAATCTA and 5′-GTGCGAGTAGAGAACGTTAAA, respectively. The scrambled control sequence is 5′-CCTAAGGTTAAGTCGCCCTCG. By blast search, this scramble sequence does not match with any sequences of mouse genes. Both the targeting and the scramble sequences were also cloned into pCAG-miR30 system (Addgene), which is a pri-miRNA based shRNA-expression vector contributed by Connie Cepko [30]. Conditional expression of GFP were performed using plasmid pCAG-ERT2CreERT2 and pCALNL-GFP [30] (Addgene). pCALNL-GFP contains a loxp-neostop-loxp sequence upstream of GFP that can be induced to express by Cre-mediated DNA recombination. Expression of Cre from pCAG-ERT2CreERT2 is controlled by Tamoxifen injection.

Mouse *Foxp1* was analyzed for potential CRISPR/Cas9 targets in silico by CRISPR gDNA design tool DNA2.0 at https://www.dna20.com/eCommerce/cas9/input. The targeting sequence with the highest predicted on-target score (100) against *Foxp1* (TGTATCATTCGTACCTCTTT) plus the NGG was synthesized and subcloned into pX330 vector (Addgene plasmid #42230) [31]. The vector pX330 was taken as the control that expressed Cas9 nuclease without *Foxp1* targeting sequence. All constructs were verified by sequencing and purified using EndoFree plasmid maxi kit (Qiagen).

### Primary cortical neuron culture

Mouse fetal brains were isolated at embryonic day 14.5 (E14.5) by dissection and placed into chilled (4°C) HBSS solution enriched with 25 mM HEPES (Invitrogen). Cortical tissues from dorsal telencephalon were dissociated with 0.05% trypsin (Invitrogen) for 10 min at 37°C and then triturated with fire-polished glass pipettes. The cell suspension was passed through a cell strainer and resuspended in DMEM (Invitrogen) supplemented with 10% FBS (Gibico). *Foxp1* shRNAs were transfected into the primary cortical cells by Nucleofector (Amaxa Biosystems, Cologne, Germany), according to the manufacturer’s protocol. Then, the cells were plated onto poly-ornithine (Sigma, 0.001%) and laminin (Invitrogen, 5 mg/ml)-coated coverslips and cultured in DMEM supplemented with 10% FBS. Following cell attachment, the culture medium was replaced with Neurobasal (Invitrogen) in the presence of 1% penicillin—streptomycin and 2% B27 supplement (Gibico). After culture *in vitro* for two days, the cells were collected to measure the expression of *Foxp1* by quantitative PCR.

### N2a Cell Culture and Real-time PCR

Neuroblastoma N2a cells were cultivated in MEM supplemented with 1% glutamine, 10% FBS, and 1% antibiotics. Efficiency of *Foxp1* knockdown with shRNAs was tested by transiently transfecting N2a cells with 1μg of shRNAs using Lipofectamine 2000 (Invitrogen). After 48h, mRNA was isolated from independent samples using the RNeasy method (Invitrogen). After conversion into cDNAs using a SuperScript First-Strand cDNA Synthesis kit (Invitrogen), quantitative RT-PCR was performed in a 96-well plate using an ABI 7500HT instrument. The relative expression of *Foxp1* was analyzed by the ΔΔCt method (where Ct is the threshold cycle number) using *Gapdh* as a housekeeping gene.

### Western blotting

Proteins from lysed N2a cells were denatured and loaded on sodium dodecyl sulfate polyacrylamide gels. After electrophoresis, the separated proteins were transferred to PVDF membranes, blocked in TBS-T (150 mM NaCl, 10 mM TRIS-HCl pH 7.5, and 0.1% Tween 20) containing 5% (w/v) dry milk, and stained with anti-FOXP1 (1:1000, Abcam) and anti-GAPDH (1:500, Santa Cruz) antibodies. The specific bands were analyzed by immunoblot infrared imaging system (LI-COR Biosciences) after incubation with the corresponding secondary antibodies. The density of each band was measured using NIH ImageJ and subtracted from the nearby background.

### *In utero* electroporation (IUE)

To study the effects of *Foxp1* knockdown or overexpression, we used *in utero* electroporation (IUE) to reliably deliver plasmid DNA into the somatosensory cortex. *In utero* electroporation was performed as described previously [32,33]. Briefly, timed-pregnant female mice were anesthetized by intraperitoneal injection of ketamine (100mg/kg) and xylazine (10mg/kg) diluted in sterile 0.9% saline. The abdomen was cleaned with 70% ethanol. A laparotomy of 3cm incision was performed in the low middle abdomen, and the uterus was carefully taken out. Approximately 3μg of indicated plasmid mixed with 1μg of GFP-pCAGGS and 0.025% of Fast-Green was delivered into the lateral ventricles of the embryos. Electric pulses (40 mV for 50 ms) were applied to the brains five times at intervals of 950 ms with an electroporator (BTX, T830). Upon completion of injection and electroporation, the uterus was then placed back into the abdominal cavity, and the abdomen wall and skin were sutured. The pregnant mouse was placed on a heating pad until it became conscious, and the embryos were allowed to develop *in utero* until when ready for the measurements.

### Immunostaining for migration and morphology analysis

Transcardial perfusion with 4% paraformaldehyde (PFA) was performed on the mice for fixation. The brains of E17.5, P0, P2, P4, P7, P14, P30 mice were dissected and post-fixed at 4°C with 4% PFA for 2h to overnight, depending on the size of brains. The brains were dehydrated in 30% sucrose and embedded in OCT compound. Cryosections were cut at 14-μm, or 80-μm thickness with a Cryostat (HM505E, Microm, Germany). Immunostaining was performed with standard protocols: brain sections were incubated overnight with primary antibodies at 4°C and incubated with appropriate fluorescent secondary antibodies for 2h at room temperature. The following primary antibodies were used: Goat anti-GFP (1:1000, Novus Biologicals); Mouse anti-FOXP1 (1:125, Abcam); Rabbit anti-Tbr2 (1:300, Abcam); Rabbit anti-Tbr1 (1:200, Abcam); Mouse anti-Satb2 (1:100, Santa Cruz); Mouse anti-Nestin (1:200, Abcam); Rabbit anti-CDP (1:50, Santa Cruz); Rat anti-Ctip2 (1:500, Abcam); Mouse anti-phospho Histone H3(1:300, Abcam); Mouse anti-β-III-tubulin (1:500, Promega).

Immunofluorescence images were obtained using either Olympus BX41 or Zeiss LSM 710 confocal microscope. The morphology of neurons in the cortex or culture was traced and analyzed using Neurolucida software (MBF Bioscience).

### Cell counting and Statistics

Cortical subregions (SVZ/VZ, IZ, and CP) were identified based on cell density using DAPI staining [34]. Brains or slices from at least three independent experiments were processed for each experimental condition. All data were presented as mean ± SEM. Statistical significance was determined using an unpaired Student’s *t-*test or one-way ANOVA, and differences were considered significant at a level of p<0.05.

## Results

### Knockdown of *Foxp1* delays neuronal radial migration in developing cerebral cortex

**Fig 1:**
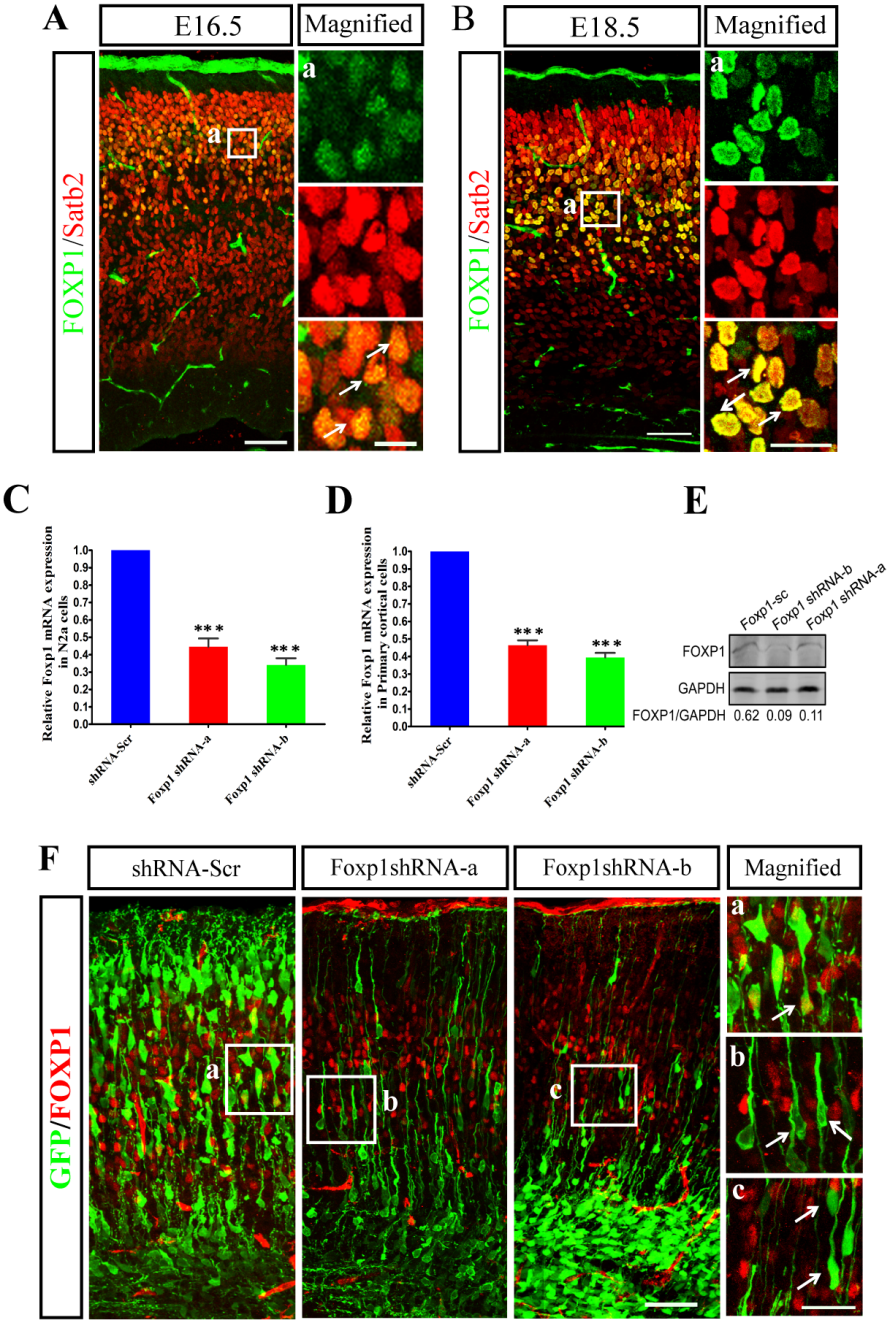
*Foxp1* is expressed at CP in mouse embryonic brain and repressed by shRNA constructs. Brain sections (14 μm) obtained from E16.5 (A) and E18.5 (B) mice were immunostained with antibodies against FOXP1 (green) and Satb2 (red). a, High-magnification images of the boxed regions in the left panels. Scale bar, 20μm in A, 50μm in B. (C) and (D), Relative mRNA expression of *Foxp1* in N2a cells (C) and in cultured cortical cells (D) transfected with shRNA-Scr or two *Foxp1* shRNAs (shRNA-a and shRNA-b). ***p<0.001, one-way ANOVA. (E), GFP-pCAGGS was cotransfected into N2a cells with shRNA-Scr, *Foxp1* shRNA-a or shRNA-b. After 48h, cells were collected and analyzed for the expression of FOXP1 and GAPDH by western blot. The expressions of FOXP1 were quantitated as rations of the background-subtracted intensities for FOXP1 to GAPDH. (F), GFP-pCAGGS was electroporated with shRNA-Scr, *Foxp1* shRNA-a or shRNA-b into cerebral cortices at E14.5. Coronal sections were obtained from the cerebral cortices at E17.5 and immunostained with anti-GFP (green) and anti-FOXP1 (red). a, b, c, High-magnification images of the boxed regions in the left panels. Arrows indicate GFP-positive neurons. Scale bar, 50μm in F, 20μm in c.

Expression of mouse *Foxp1* was first detected in the cortical plate at E14.5 [16]. To investigate the potential functions of *Foxp1* in corticogenesis, we characterized its expression in embryonic cortices by immunofluorescence. Consistent with the previous study [16], FOXP1 protein was localized in the developing cortical plate but not in the IZ and VZ/SVZ at E16.5 and E18.5 (Fig 1A and 1B). Most *Foxp1*-expressing cells in the CP co-expressed with Satb2, a marker of callosal projection neurons in the upper layers (Fig 1A and 1B). The results suggest that *Foxp1* is expressed by the early post-mitotic neurons in developing cortical plate.

To explore the function of *Foxp1* in cortical development, we sought to decrease the endogenous expression of *Foxp*1 by RNA interference. Two short hairpin RNA constructs designed against *Foxp1*, and a scrambled shRNA plasmid as a control that does not target to any mouse gene were tested in mouse neuroblastoma N2a cells and cultured cortical cells. qRT-PCR analysis showed that the *Foxp1* mRNA level was significantly decreased after transient transfection with the plasmids expressing *Foxp1* shRNA-a and shRNA-b as compared to that expressing the control (shRNA-Scr) (Fig 1C and 1D). Western blot analysis revealed that both shRNA-a and shRNA-b dramatically decreased the FOXP1 expression (Fig 1E). To confirm the efficiency of RNAi on the inhibition of FOXP1 in pyramidal neurons, the plasmids were delivered into the lateral cortices of E14.5 embryos by *in utero* electroporation. Embryonic brains receiving either a *Foxp1* shRNA or shRNA-Scr along with the GFP-pCAGGS were harvested three days after the electroporation. Immunofluorescence showed that a sizable percentage of scramble RNA-transfected cells was FOXP1 positive, whereas few cells receiving shRNAs expressed FOXP1 (shRNA-Scr: 32.0%; shRNA-a: 2.6%; shRNA-b: 3.6%) (Fig 1F), suggesting the knockdown of *Foxp1 in vivo*.

**Fig 2:**
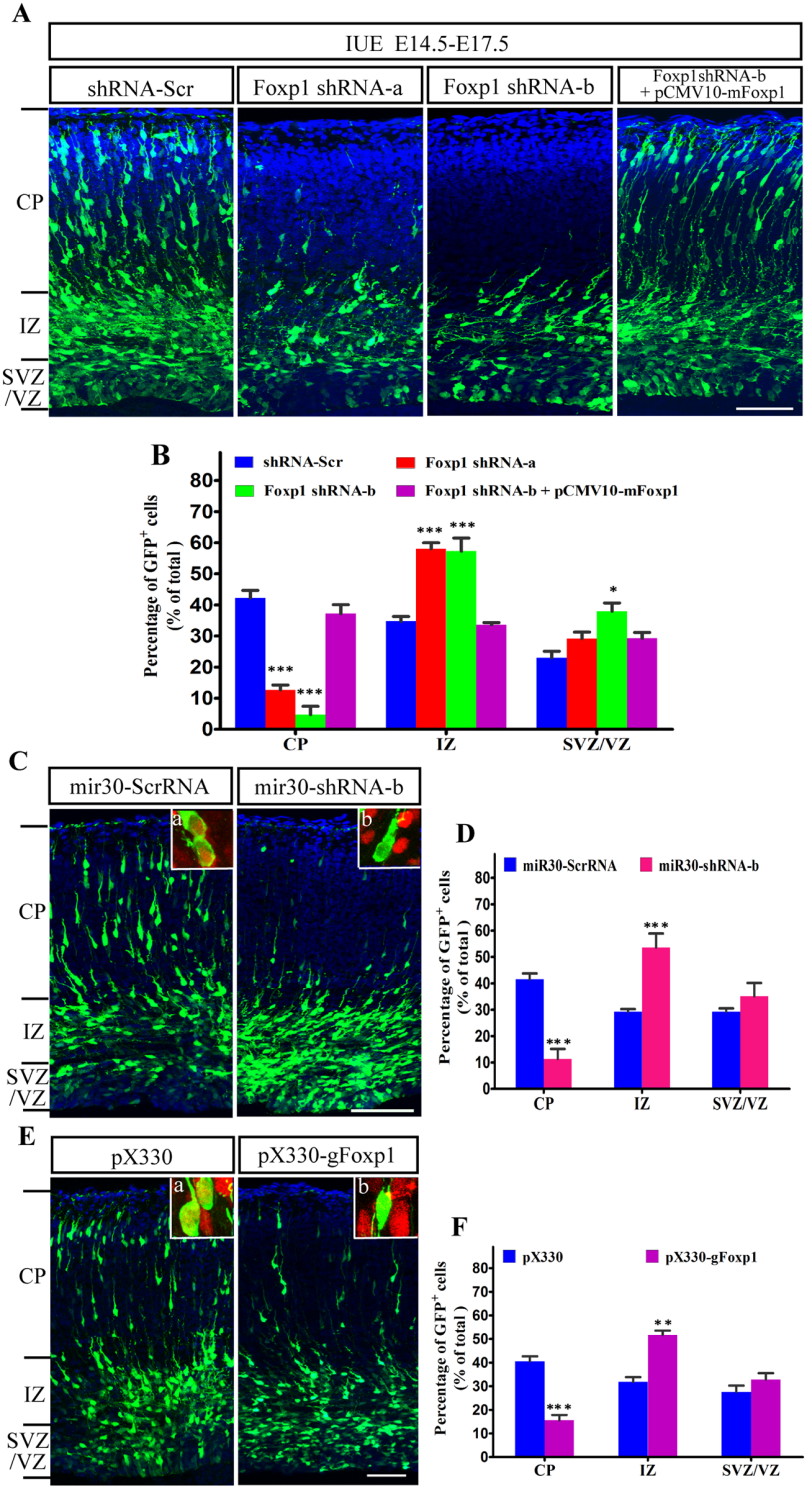
Suppression of *Foxp1* inhibits neuronal migration. (A), E14.5 mouse embryos electroporated with a GFP expression plasmid along with shRNA-Scr, *Foxp1* shRNA-a or shRNA-b together with pCMV-mFoxp1 were allowed to develop until E17.5. Coronal sections (14 μm) of E17.5 brains were immunostained with an antibody against GFP (green). Nuclei were stained with DAPI (blue). Short black lines indicate the borders between CP, IZ, SVZ/VZ. Scale bar, 50μm. (B), Statistical analysis of the percentages of GFP-positive cells in the indicated regions of the cerebral cortex as showed in (A). *p<0.05, ***p<0.001, one-way ANOVA. (C), E14.5 mice receiving mir30-ScrRNA or mir30-shRNA-b were examined at E17.5. Scale bar, 50μm. (C)-a and (C)-b, Representative images of migrating neurons (Green) stained with FOXP1 antibody (Red) showed FOXP1 expression in control neurons but not in mir30-shRNA-b expressing neurons. (D), Statistical analysis of the percentages of electroporated cells in the indicated regions of the cerebral cortex as showed in (C). ***p<0.001, Student’s *t*-test. (E), GFP-pCAGGS was coelectroporated with pX330 or pX330-gFoxp1 into E14.5 mouse brains. Sections were examined at E17.5. Scale bar, 50μm. Immunostaining for FOXP1 (Red) indicated a lack of FOXP1 expression in pX330-gFoxp1 transfected neurons (E-b). Vector pX330 transfected neurons expressed FOXP1 in the CP normally (E-a). (F), Quantitative analysis of the distribution of electroporated cells in cortical layers as showed in (E). **p<0.01 and ***p<0.001, Student’s *t*-test.

Mutations of *FOXP1* have been found in patients with global developmental delay, intellectual disability, and autism spectrum disorders. To explore the functions of *Foxp1*, we delivered *Foxp1* shRNA into the somatosensory cortex, deficits of which have been observed in many developmental neuronal disorders [35]. The developing cortical neurons were transfected at E14.5 with a scramble or *Foxp1* shRNAs, and analyzed at E17.5 in the cortical wall that was regionally divided into CP, IZ, and SVZ/VZ (Fig 2A). As shown in Fig 2B, knocking down *Foxp1* resulted in accumulation of electroporated cells in IZ (shRNA-Scr: 34.7%; shRNA-a: 58.0%; shRNA-b: 57.3%) and a decrease of the neurons in CP as compared to the control experiments (shRNA-Scr: 42.3%; shRNA-a: 12.7%; shRNA-b: 4.7%). In addition, more GFP-positive cells in SVZ/VZ were found in the brains transfected with *Foxp1* shRNAs than that with the control. A significant retention in SVZ/VZ was induced by the potent *Foxp1* shRNA-b (shRNA-Scr: 23.0%; shRNA-a: 29.2%; shRNA-b: 37.9%), indicating a migratory delay. To confirm that the migration defect caused by *Foxp1* shRNAs is due to *Foxp1* deficiency, we performed a rescue experiment by over-expression of a functional *Foxp1* (pCMV10-mFoxp1) together with shRNA-b. As shown in Fig 2B, the distribution of neurons transfected with pCMV10-mFoxp1 plus shRNA-b was almost identical to that of the control group (Fig 2B). Introduction of a plasmid overexpressing *Foxp1* alone did not induce apparent neuronal migration anomalies since a similar population of GFP-positive cells reached the superficial layers three days after transfection (data not shown). These results indicate that endogenous *Foxp1* depletion hindered the radial migration of cortical neurons.

To exclude possible off-targeting effects of vector-driven shRNA, we employed two different strategies to inhibit the *Foxp1* specifically. First, the expression of targeting sequences from pri-miRNA undergoes more natural microRNA processing and does not produce any off-target effect [36]. Therefore, the targeting and scramble sequences were embedded into the murine miR-30 using pCAG-miR30 vector system. The miR30-based shRNA expression system was introduced into the brain by IUE at E14.5. At E17.5, a reproducible migration defect was observed in *Foxp1* miR30-shRNA-b group by comparison with the control (miR30-ScrRNA) (Fig 2C). The miR30-shRNA-b transfected cortex had a decreased population of the cells in CP when compared with the scrambled control (miR30-ScrRNA: 41.6%; miR30-shRNA-b: 11.3%). Correspondingly, more neurons were stalled in the IZ when *Foxp1* was inhibited (miR30-ScrRNA: 29.2%; miR30-shRNA-b: 53.5%) (Fig 2D), indicating a migratory delay. Next, to further prove the specificity, the fourth exon of mouse *Foxp1* was targeted for disruption by CRISPR/Cas9-mediated genetic editing. pX330-gFoxp1 together with GFP-pCAGGS were electroporated into E14.5 brains. A previous study had showed the effectiveness of pX330 in driving gene knockdown in post-mitotic neurons following IUE [37]. Compared to the control (CP: 40.6%; IZ: 31.9%), only 15.6% of pX330-gFoxp1 transfected neurons entered the CP while 51.7% remained in the IZ (Fig 2E and 2F). Both miR30-shRNA and pX330-gFoxp1 inhibited the expression of FOXP1 in the E17.5 neurons as demonstrated by the loss of colocalization of GFP and FOXP1 immunofluorescence in the CP (Fig 2Ca–b and 2Ea–b). Taken together, by targeting to two different regions of the gene at RNA and genomic levels, these results reveal that the migration defect was unlikely due to off-target effects but caused by the specific down-regulation of *Foxp1*. This conclusion was further supported by preliminary stuidies on the expression of upper layer marker Cux1 in the mice with a brain-specific deletion of *Foxp1* [20]. In contrast to the wild-type where most Cux1^+^ cells appeared only in the layer II-IV, many Cux1^+^ cells were present in the deep layer V and VI of the knockout mice during the early postnatal development (Data not shown).

**Fig 3:**
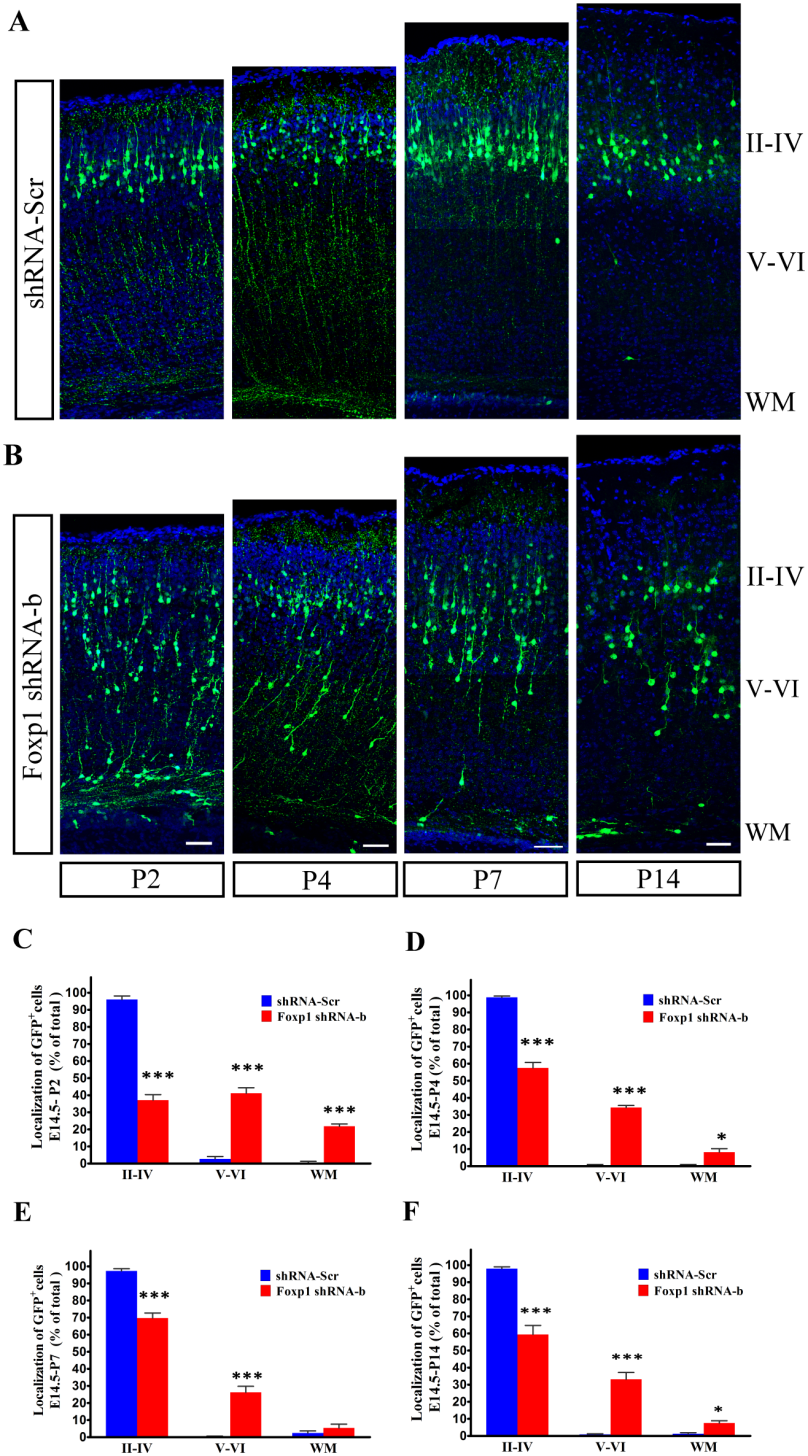
Effects of *Foxp1* knockdown on the placement of cortical neurons. (A), (B) E14.5 embryos were electroporated with the control or *Foxp1* shRNA-b, together with GFP-pCAGGS, and analyzed at P2, P4, P7, and P14. Scale bar, 50μm. (C), (D), (E), (F) Histograms showed the percentage of transfected cells in different regions of the cerebral cortex at P2, P4, P7, and P14. *p<0.05 and ***p<0.001 for comparisons between shRNA-b and the corresponding control, Student’s *t*-test.

To investigate the long-term effects of knocking down *Foxp1* on neuronal migration, we electroporated shRNA-b into VZ cells at E14.5 and determined the positions of GFP-positive cells at different time points during postnatal development (Fig 3A and 3B). At P2, a time point by which cortical neurons have almost completed radial migration in normal condition; most control plasmid transfected neurons migrated to the cortical layer II-IV (96.0%). By contrast, only 37.2% of shRNA-b transfected neurons were in layer II-IV, whereas a large number of GFP-positive neurons ectopically located in layer V-VI (41.1%) and the white matter (WM) (21.8%) (Fig 3C). Thus, the defect in migration of *Foxp1* knockdown cells was not temporary but persisted after six days of electroporation. Moreover, the aberrant positioning of shRNA-b transfected neurons in the CP were also observed at P4, P7, and P14, suggesting that the migratory defects cannot be attributed to a developmental delay (Fig 3D–3F). Together, these data imply that the disruption of *Foxp1* has a significant impact on the neuronal placement in the cerebral cortex.

### *Foxp1* knockdown does not affect fate determination, proliferation and differentiation of cortical neurons

**Fig 4:**
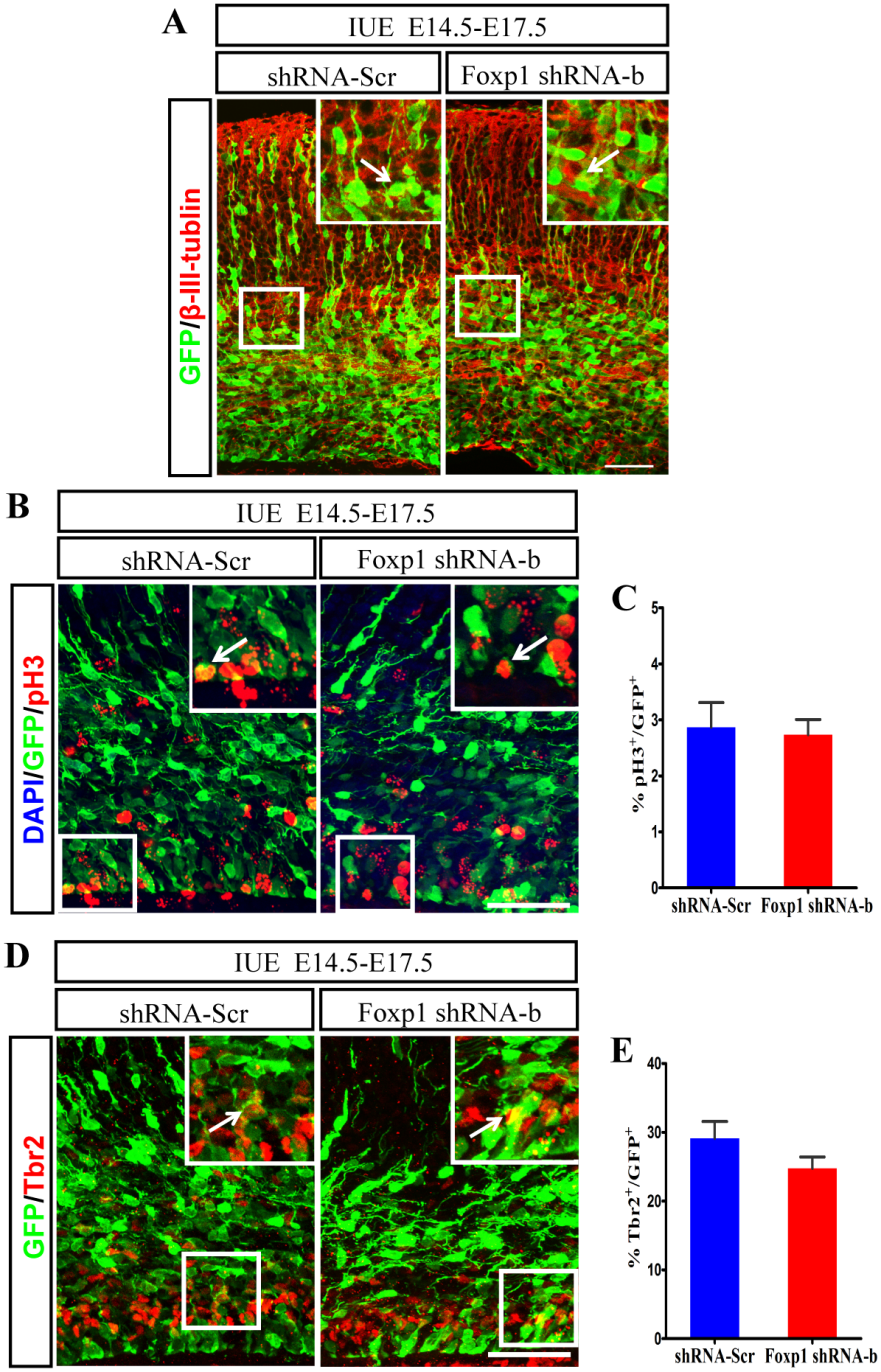
*Foxp1* insufficiency does not affect cell division and specification of neuronal progenitors. *Foxp1* shRNA-b or shRNA-Scr was transfected into progenitor cells of the mouse cortex at E14.5 by IUE, and brains were fixed at E17.5. Coronal sections were immunostained for neuronal marker β-III-tubulin (A), M-phase marker pH3 (B), intermediate/basal progenitor marker Tbr2 (D). (C), Quantification of the percentage of pH3 positive, GFP-positive neurons. (E), Quantification of the percentage of Tbr2 positive, GFP-positive neurons in the SVZ/VZ of the cerebral cortex. Scale bar, 50μm in A, B and D. Student’s *t*-test.

To examine whether the shRNA-electroporated cells blocked in the IZ adopt a correct neuronal fate, we stained the brain sections at E17.5 with antibody against β-III-tubulin (a neuronal marker). *Foxp1* knockdown cells expressed β-III-tubulin normally as those cells receiving the control shRNA (Fig 4A), suggesting the stalled cells in the IZ already exited from the last mitosis and became neurons. Consistently, only a small proportion of transfected cells remained to be mitotic, as recognized by immunostaining of phosphor-Histone, pH3. The ratios of pH3 and GFP double positive cells in the total GFP-positive population were 2.7% in the control and 2.9% in the shRNA-b transfected group (Fig 4B and 4C). This indicates that *Foxp1* knockdown did not alter the mitotic activity of cells in the VZ and SVZ. To analyze the specification of neuronal progenitors, the brain sections were examined for the expression of Tbr2 (intermediate/basal progenitor marker) three days following IUE. The percentage of the electroporated cells labeled with Tbr2 in the SVZ/VZ did not have a significant difference between the control and *Foxp1* knockdown group (shRNA-Scr: 29.1%; shRNA-b: 24.7%) (Fig 4D and 4E). Further, to exclude the possible contribution of cell death to the abnormal migration, we examined the brain sections with an apoptotic marker, the cleaved caspase-3(CC3). No significant change was detected in the proportions of the apoptotic cells. There was an average of 0–2 apoptotic cells per section in both control and *Foxp1* knockdown brains (data not shown).

**Fig 5:**
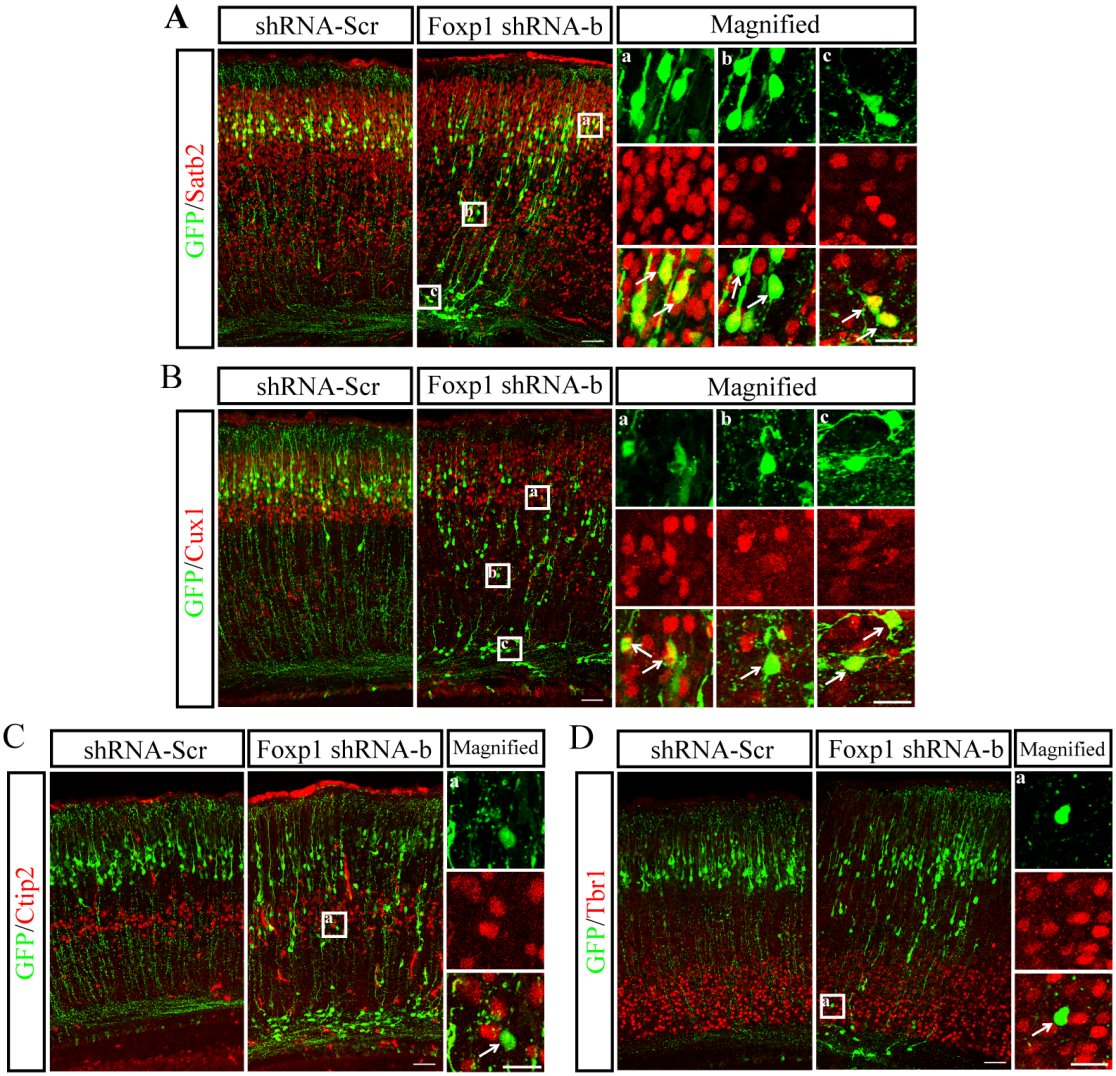
*Foxp1* knockdown does not affect neuronal differentiation. E14.5 mouse embryos were electroporated with *Foxp1* shRNA-b or shRNA-Scr, and brains were fixed at P2. Coronal sections were immunostained for Satb2 (A) and Cux1 (B). a, b, c, High-magnification images of the boxed regions in the left panels. a, b, c in (A) and (B) represent layer II-IV, layer V and white matter, respectively. (C), (D), Immunofluorescence staining of Ctip2 and Tbr1 in brain sections from control and *Foxp1* shRNA-b. (C)-a, (D)-a, High-magnification images of the boxed regions in the left panels. Scale bar, 50μm in (A), (B), (C) and (D).

To determine whether this perturbation of neuronal localization was induced by aberrant differentiation of cortical progenitors, we examined the expression of cell type-specific markers (Cux1, a marker of layer II-IV neurons; Satb2, a marker of callosal projection neurons in the upper layers; Ctip2, a marker of layer V pyramidal neurons; Tbr1, a marker of layer VI neurons) in GFP-positive neurons at P2. As illustrated in Fig 5A and 5B, most *Foxp1* knockdown cells in the CP correctly expressed Satb2 and Cux1, similar to those in the control group. The GFP-positive neurons stalled in the deep layers weakly expressed Satb2 and Cux1 but not Ctip2 and Tbr1, suggesting that they were not the typical neurons in layer V and VI (Fig 5C and 5D). These data demonstrate that *Foxp1-*downregulated cells share the molecular characteristics of the upper layer cortical neurons, despite their aberrant positions.

### *Foxp1* knockdown disrupts early neurite development

**Fig 6:**
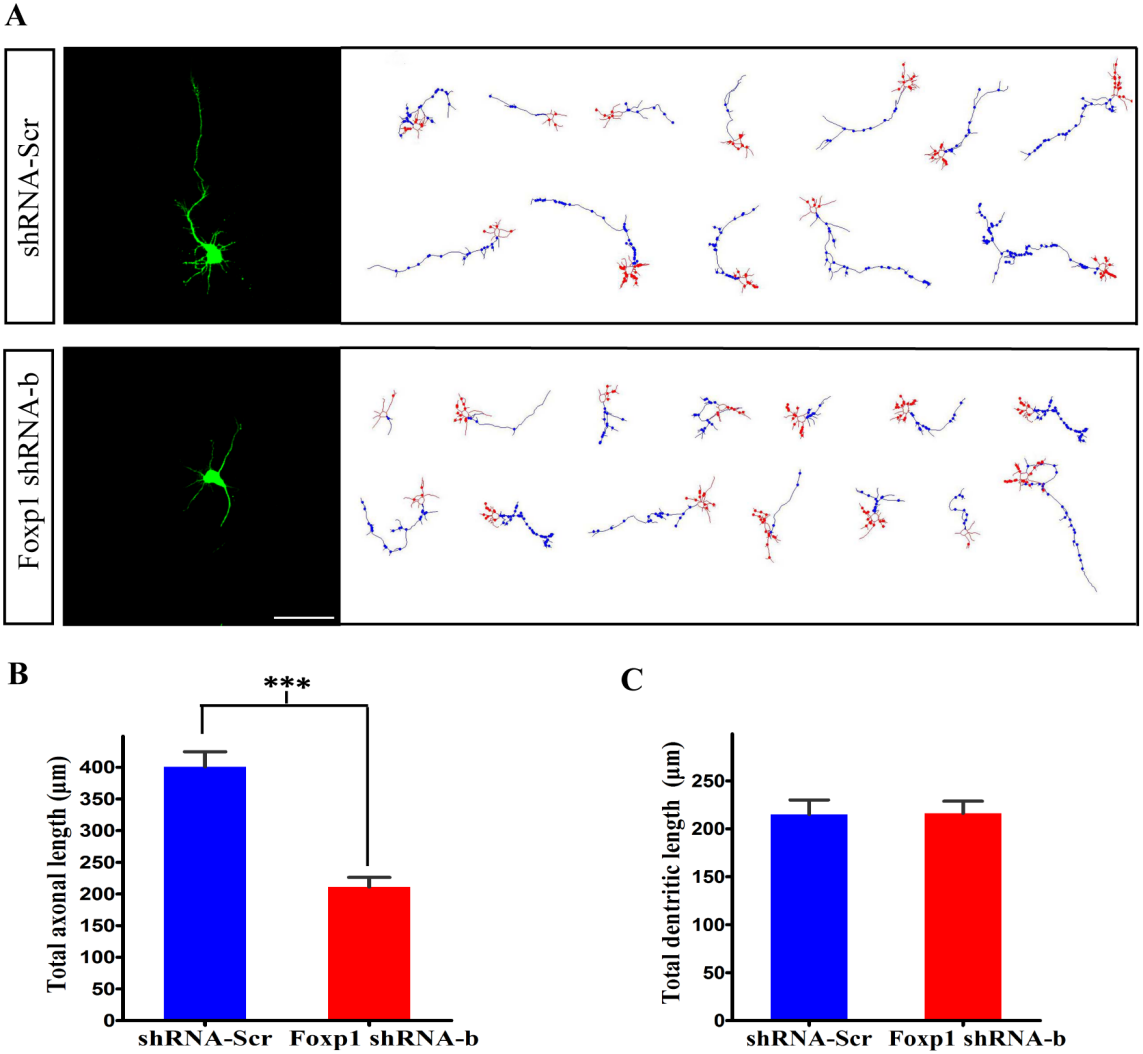
*Foxp1* regulates neuronal morphogenesis *in vitro*. (A), Cortical neurons were transfected *in utero* at E14.5 and isolated at E15.5 for primary cultures *in vitro*. At day four *in vitro*, the neurons transfected with GFP-pCAGGS together with shRNA-Scr (n = 39) or *Foxp1* shRNA-b (n = 53) were analyzed by Neurolucida. Scale bar, 50μm. (B), Quantitative analysis of total lengths of the axon and dendrites from images in (A). ***p<0.001, Student’s *t*-test.

To investigate the possible mechanisms underlying the migration defect, we first examined the morphology changes of neurite outgrowth *in vitro*. Cortical neurons were isolated one day after *in utero* electroporation at E14.5 with GFP-pCAGGS together with either the control shRNA or *Foxp1* shRNA-b. These neurons were maintained in culture for 4 days and then fixed. The morphology of individual GFP-positive neurons was imaged by confocal microscopy and analyzed using Neurolucida. The majority of GFP-positive neurons in the control shRNA group had a typical polarity: a single, long axon and multiple shorter dendrites (Fig 6A). Quantitative analysis revealed that the complexity and the total length of dendrites did not differ between *Foxp1* knockdown and the control neurons (Fig 6C). However, *Foxp1* knockdown led to a remarkable reduction in the total length of axons as compared with the control (shRNA-Scr: 400.5μm; shRNA-b: 210.8μm) (Fig 6B). Indeed, on transfection of *Foxp1* shRNA-b constructs, the length of axons in a few of the GFP-labeled neurons was virtually equivalent to that of the dendrites. These data demonstrate an essential role of *Foxp1* in regulating axonal elongation and neuronal polarity *in vitro*.

**Fig 7:**
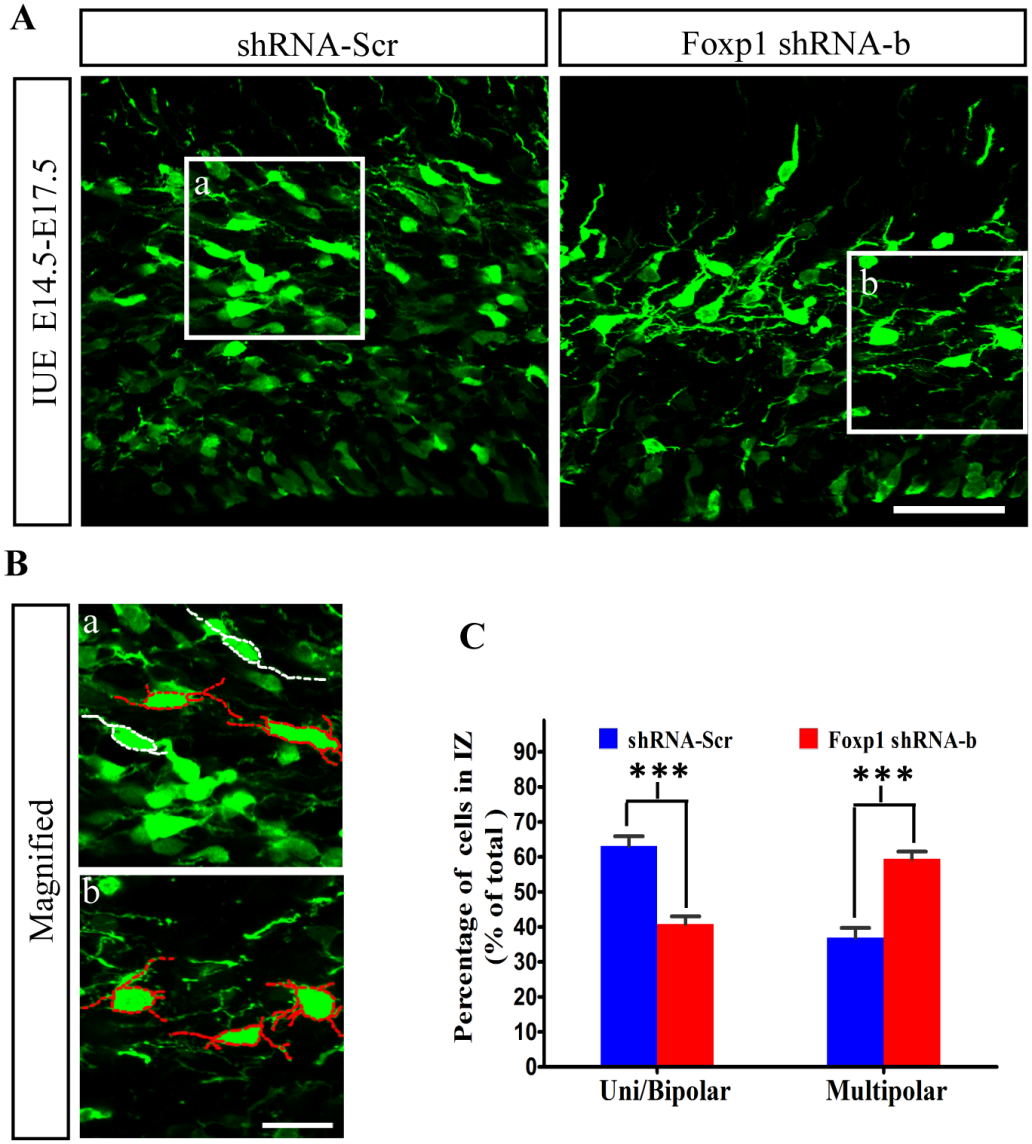
Multipolar to bipolar transition of migrating neurons is compromised by *Foxp1* knockdown. (A), Brains were electroporated *in utero* with control shRNA or shRNA-b at E14.5 and examined at E17.5. (B), Representative GFP-labeled neurons in the IZ region in each group. White and red lines show cells with uni/bipolar and multipolar morphologies, respectively. (C), Quantitative and statistical analysis of the percentages of uni/bipolar and multipolar neurons in the IZ. Scale bar, 50μm in (A) and 20μm in (B). ***p<0.001, Student’s *t*-test.

The early neuronal transition from multipolar to bipolar morphology in the IZ is a critical point of migration control and a vulnerable target for disruption of neocortical development [3,38,39]. To confirm the importance of *Foxp1* in cortical neuronal morphogenesis *in vivo*, we determined the percentages of transfected neurons with bipolar or multipolar processes for both control and *Foxp1* shRNA-b group (Fig 7A). We found that those cells reaching CP in both groups acquired uniform bipolar morphology at E17.5. However, the percentage of bipolar cells in the IZ was substantial lower in the *Foxp1* knockdown mice (36.9%) than in the control (63.0%), indicating that *Foxp1* depletion compromises the polarization process that normally occurs for the newborn neurons in the IZ. The reduction of bipolar neurons was accompanied by an increase in the multipolar cells (Fig 7C). These results are consistent with our findings from the neuronal culture *in vitro* (Fig 6). A reduced capability in the axon outgrowth will likely hinder the bipolar formation *in vivo*. It is possible that disruption of multipolar to bipolar transition may contribute to the radial migration defects caused by knockdown of *Foxp1* gene in the embryonic cortex.

### Knocking down *Foxp1* alters dendritic morphology of mature cortical neurons

**Fig 8:**
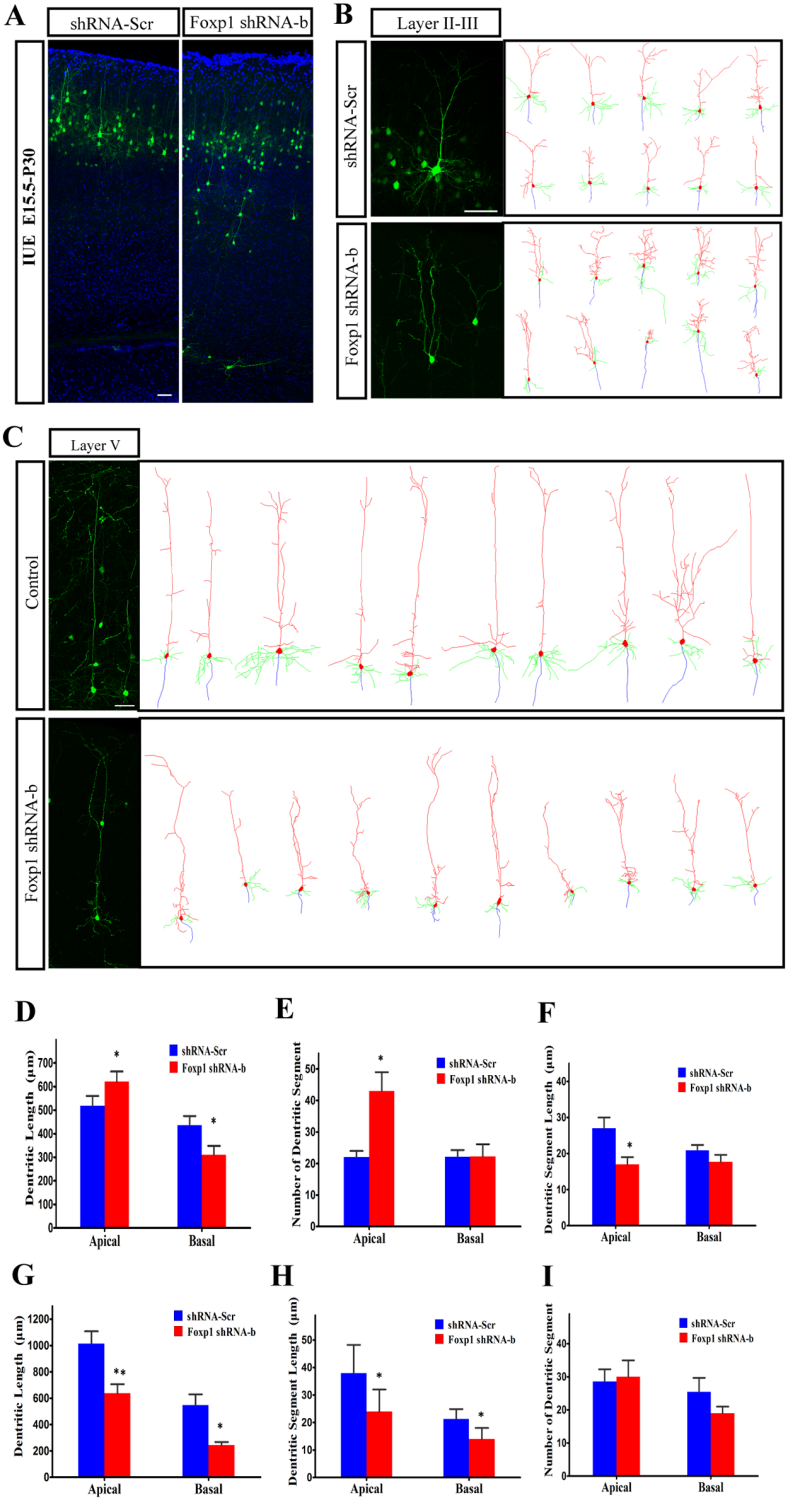
Knockdown of *Foxp1* in pyramidal neurons leads to abnormal dendritic development at P30. (A), Coronal sections (80 μm) from *Foxp1* knockdown and the control mice. Scale bar: 50μm. (B), Representative images and Neurolucida tracings of GFP-positive, layer II-III neurons from the control (n = 18) and *Foxp1* knockdown (n = 17) mice at E15.5. Scale bar: 50μm. (C), Representative images and Neurolucida tracing of GFP-positive neurons stalled in layer V (n = 15) from shRNA-b transfected mice at E15.5 or from the control (n = 11) transfected mice at E13.5. (D), (E), (F), Quantitative and statistical analysis of dendritic parameters in layer II-III. (G), (H), (I), Quantitative and statistical analysis of dendritic parameters in layer V at P30. *p<0.05, **p<0.01, Student’s *t*-test.

To further confirm the effects of *Foxp1* knockdown on neuronal morphology, dendrite formation was examined in mature cortical pyramidal neurons. To improve the separation of single neurons for morphological analysis, we utilized a conditional Cre/loxP-mediated expression system to induce the relatively sparse distribution of transfected cells that express GFP. The number and density of GFP-labeled cells were controlled by the dose and exposure time of tamoxifen. pCAG-CreERT2Cre and pCALNL-GFP (1:1 mixture) were co-transfected into the brains at E15.5, along with either *Foxp1* shRNA-b or shRNA-Scr, by *in utero* electroporation. Electroporation at E15.5 allows for transfection of only external pyramidal neurons in layer II-III without labeling the spiny cells in layer IV, thus simplifying the morphological comparison between the two groups. The expression of GFP was initiated at P22 by intraperitoneal injection of tamoxifen. Brains of P30 mice were collected and processed. As shown in Fig 8A, part of the *Foxp1* shRNA-b transfected neurons had abnormal placement within the laminated cortex while others reached the upper layers normally. The neurons in layer II-III assumed the characteristic pyramidal morphology in both *Foxp1*-knockdown and control group (Fig 8B) and were taken for morphological analysis using Neurolucida. Quantitative analysis found that loss of *Foxp1* significantly increased the number of dendritic segments and total length of the apical dendrites (Fig 8D and 8E), whereas the average segment length of the apical dendrites was decreased (Fig 8F). In contrast, the number of dendritic segments and the average segment length of basal dendrites were not altered although the total dendrite length was reduced in the *Foxp1* suppression neurons (Fig 8D–8F). Thus, this set of data suggests that *Foxp1* gene may be a determinant of growth and branching of apical dendrites of the cortical pyramidal neurons.

Interestingly, the *Foxp1* shRNA-b transfected neurons stalled in layer V had a remarkably different morphology as those pyramidal neurons normally in the deep layers. The normal layer V neurons were labeled by transfection with pCAG-CreERT2Cre and pCALNL-GFP at E13.5, and induced at P22 by tamoxifen. Examination of dendritic pattern at P30 demonstrated that ectopic neurons with *Foxp1* knockdown exhibited marked shorter apical and basal dendrites when compared with the normal layer V neurons (Fig 8G). In addition, mean segment length of the dendrites was significantly reduced (Fig 8H). These data suggest that the ectopic neurons may have acquired a morphology with certain similarities to the upper layer pyramidal neurons, which are shorter and smaller than those of layer V.

## Discussion

In this study, we have identified *Foxp1* as an essential transcription factor required for appropriate migration of cortical neurons. The migration defects observed at E17.5 persisted in the *Foxp1* knockdown neurons after birth, leading to inappropriate placement of electroporated cells in the cerebral cortex. *Foxp1* knockdown did not alter the neuronal identities. The apparent neuronal heterotopias at postnatal days due to *Foxp1* deficiency were similar to the phenotypes resulting from the suppression of *Doublecortin*, *Robo4*, *cKit*, *Dpy19l1* or *PHF6* in the cerebral cortex by *in utero* RNA interference [40–44]. In addition, *Foxp1* is involved in the regulation of early axonal elongation as observed in the neurons cultured *in vitro* after gene transfection *in utero*. The advantage of this experimental scheme is that a group of pyramidal neurons were selectively labeled and analyzed in cultures. Consistent with the *in vitro* observation, we have discovered that *Foxp1* has an impact on multipolar-bipolar shape conversion *in vivo*. Since the multipolar stage is a critical point of migration control [3], the migratory defect caused by *Foxp1* knockdown may result from a weakened bipolar formation of early neurons. It is well known that the mammalian cerebral cortex is the region of the brain responsible for cognitive function, sensory perception, voluntary motor control, and consciousness. The coordinated migration of neurons is essential for functional and architectural formation of the mammalian cerebral cortex. The migration errors of cortical neurons may lead to cognitive impairments and increase susceptibility to major psychiatric and neurological disorders [45–47]. A *de novo* heterozygous deletion of *FOXP1* has been found in a patient with severe speech delay, delayed motor development, and epileptiform discharges [22]. Based on the findings from this study, it is likely that the deletion of *FOXP1* may interfere with the normal localization of cortical neurons. Such kind of neuronal heterotopias may bring about the incorrect activity of cortical circuits and contribute to the pathogenesis of epileptiform discharges [48,49]. Future studies are needed to determine the electrophysiological properties and connectivity of cortical neurons with haploinsufficiency of functional *Foxp1*.

The long-term effect of *Foxp1*-knockdown on morphology of mature neurons was evaluated here by a conditional inducible system. Cre-loxp inducible expression system in combination with *in utero* electroporation allow us to minimize the number of cells expressing GFP at specific time points so that the morphology of transfected neurons could be better visualized and analyzed. Since axons become tangled in the corpus callosum and were truncated after the tissue sectioning, we cannot measure the length of individual axons. However, the morphology of dendritic trees in the mature neurons was successively quantitated. It was found that neurons in layer II-III with *Foxp1* knockdown have an increase in the branching orders and a decrease in the segment length of the apical dendrites (Fig 8). The disturbance of dendrite architecture in the upper layers may lead to the pathology of mental retardation and cognition-affecting neurological diseases [50–52]. Haploinsufficiency of *FOXP1*, either from an intragenic deletion, N-terminal deletion, a premature translational stop, a reading frame shift or other protein altering mutations has been found in cohorts of patients with intellectual disability and autism spectrum disorders [23–25,27]. It is possible that the aberrant dendritic structure from the loss of *Foxp1* may contribute to the etiology of these developmental disorders.

The mechanisms of action by *Foxp1* remain to be determined. Interestingly, FOXP1 is co-localized with its closely related family member FOXP2 in several brain regions of the bird, mouse, and human brain [16,53]. Clinical studies illustrate that disruptions of *FOXP1* and *FOXP2* in human could have overlapping phenotypes including the neurodevelopment disorders [21]. FOXP1 protein interacts with FOXP2 and FOXP4 to form a homo-, or hetero-dimer, which controls transcription of multiple downstream target genes [54,55]. Although many target genes of *Foxp2* have been identified through high-through analysis of promoter occupancy [56,57], the precise target mediating the neuronal function of *Foxp1* in the cerebral cortex is not clear. *Foxp2* regulates a gene network implicated in neurite outgrowth in the developing brain [58]. This gene network may provide a clue to delineate the *Foxp1* targets that modulate the morphogenesis of cortical neurons. In addition, a recent study found that FoxP1 stimulates angiogenesis by repressing semaphorin 5B (Sema5B) [59]. Since Sema5B is an inhibitory guidance cue for neuronal axons and is expressed in the cerebral cortex [60,61], this finding presents a possibility that *Sema5B* may be a target gene of *Foxp1* for regulating the cortical development. It will be interesting to determine whether *Foxp1* represses the expression of *Sema5B* in the cortical neurons.

In sum, we have delineated *Foxp1* as a molecular determinant of radial neuronal migration and morphogenesis. These results not only shed light on the development of the cerebral cortex; they might also represent an interesting entry point for dissecting the molecular mechanisms underlying certain neurodevelopmental disorders such as intellectual disability, autism, and language impairment.

## References

[1] Sidman RL, Rakic P. Neuronal migration, with special reference to developing human brain: a review. Brain research. 1973;62(1):1–35. 4203033 10.1016/0006-8993(73)90617-3

[2] Noctor SC, Martinez-Cerdeno V, Ivic L, Kriegstein AR. Cortical neurons arise in symmetric and asymmetric division zones and migrate through specific phases. Nature neuroscience. 2004;7(2):136–44. 14703572 10.1038/nn1172

[3] LoTurco JJ, Bai J. The multipolar stage and disruptions in neuronal migration. Trends in neurosciences. 2006;29(7):407–13. 16713637 10.1016/j.tins.2006.05.006

[4] Hatanaka Y, Yamauchi K. Excitatory cortical neurons with multipolar shape establish neuronal polarity by forming a tangentially oriented axon in the intermediate zone. Cerebral cortex. 2013;23(1):105–13. 10.1093/cercor/bhr383 22267309

[5] Nishimura YV, Sekine K, Chihama K, Nakajima K, Hoshino M, Nabeshima Y, et al Dissecting the factors involved in the locomotion mode of neuronal migration in the developing cerebral cortex. The Journal of biological chemistry. 2010;285(8):5878–87. 10.1074/jbc.M109.033761 20022952 PMC2820813

[6] Noctor SC, Flint AC, Weissman TA, Wong WS, Clinton BK, Kriegstein AR. Dividing precursor cells of the embryonic cortical ventricular zone have morphological and molecular characteristics of radial glia. The Journal of neuroscience: the official journal of the Society for Neuroscience. 2002;22(8):3161–73. 11943818 10.1523/JNEUROSCI.22-08-03161.2002 PMC6757532

[7] Jossin Y, Cooper JA. Reelin, Rap1 and N-cadherin orient the migration of multipolar neurons in the developing neocortex. Nature neuroscience. 2011;14(6):697–703. 10.1038/nn.2816 21516100 PMC3102785

[8] Miyoshi G, Fishell G. Dynamic FoxG1 expression coordinates the integration of multipolar pyramidal neuron precursors into the cortical plate. Neuron. 2012;74(6):1045–58. 10.1016/j.neuron.2012.04.025 22726835 PMC3653132

[9] Ohshima T, Hirasawa M, Tabata H, Mutoh T, Adachi T, Suzuki H, et al Cdk5 is required for multipolar-to-bipolar transition during radial neuronal migration and proper dendrite development of pyramidal neurons in the cerebral cortex. Development. 2007;134(12):2273–82. 17507397 10.1242/dev.02854

[10] Pacary E, Heng J, Azzarelli R, Riou P, Castro D, Lebel-Potter M, et al Proneural transcription factors regulate different steps of cortical neuron migration through Rnd-mediated inhibition of RhoA signaling. Neuron. 2011;69(6):1069–84. 10.1016/j.neuron.2011.02.018 21435554 PMC3383999

[11] Hu H, Wang B, Borde M, Nardone J, Maika S, Allred L, et al Foxp1 is an essential transcriptional regulator of B cell development. Nature immunology. 2006;7(8):819–26. 16819554 10.1038/ni1358

[12] Shi C, Zhang X, Chen Z, Sulaiman K, Feinberg MW, Ballantyne CM, et al Integrin engagement regulates monocyte differentiation through the forkhead transcription factor Foxp1. The Journal of clinical investigation. 2004;114(3):408–18. 15286807 10.1172/JCI21100 PMC484980

[13] Shu W, Yang H, Zhang L, Lu MM, Morrisey EE. Characterization of a new subfamily of winged-helix/forkhead (Fox) genes that are expressed in the lung and act as transcriptional repressors. The Journal of biological chemistry. 2001;276(29):27488–97. 11358962 10.1074/jbc.M100636200

[14] Shu W, Lu MM, Zhang Y, Tucker PW, Zhou D, Morrisey EE. Foxp2 and Foxp1 cooperatively regulate lung and esophagus development. Development. 2007;134(10):1991–2000. 17428829 10.1242/dev.02846

[15] Wang B, Weidenfeld J, Lu MM, Maika S, Kuziel WA, Morrisey EE, et al Foxp1 regulates cardiac outflow tract, endocardial cushion morphogenesis and myocyte proliferation and maturation. Development. 2004;131(18):4477–87. 15342473 10.1242/dev.01287

[16] Ferland RJ, Cherry TJ, Preware PO, Morrisey EE, Walsh CA. Characterization of Foxp2 and Foxp1 mRNA and protein in the developing and mature brain. The Journal of comparative neurology. 2003;460(2):266–79. 12687690 10.1002/cne.10654

[17] Morikawa Y, Komori T, Hisaoka T, Senba E. Detailed expression pattern of Foxp1 and its possible roles in neurons of the spinal cord during embryogenesis. Developmental neuroscience. 2009;31(6):511–22. 10.1159/000243715 19797899

[18] Palmesino E, Rousso DL, Kao TJ, Klar A, Laufer E, Uemura O, et al Foxp1 and lhx1 coordinate motor neuron migration with axon trajectory choice by gating Reelin signalling. PLoS biology. 2010;8(8):e1000446 10.1371/journal.pbio.1000446 20711475 PMC2919418

[19] Konstantoulas CJ, Parmar M, Li M. FoxP1 promotes midbrain identity in embryonic stem cell-derived dopamine neurons by regulating Pitx3. Journal of neurochemistry. 2010;113(4):836–47. 10.1111/j.1471-4159.2010.06650.x 20175877

[20] Bacon C, Schneider M, Le Magueresse C, Froehlich H, Sticht C, Gluch C, et al Brain-specific Foxp1 deletion impairs neuronal development and causes autistic-like behaviour. Molecular psychiatry. 2014. 10.1038/mp.2014.116 PMC4419151 25266127

[21] Bacon C, Rappold GA. The distinct and overlapping phenotypic spectra of FOXP1 and FOXP2 in cognitive disorders. Human genetics. 2012;131(11):1687–98. 10.1007/s00439-012-1193-z 22736078 PMC3470686

[22] Carr CW, Moreno-De-Luca D, Parker C, Zimmerman HH, Ledbetter N, Martin CL, et al Chiari I malformation, delayed gross motor skills, severe speech delay, and epileptiform discharges in a child with FOXP1 haploinsufficiency. European journal of human genetics: EJHG. 2010;18(11):1216–20. 10.1038/ejhg.2010.96 20571508 PMC2987472

[23] Hamdan FF, Daoud H, Rochefort D, Piton A, Gauthier J, Langlois M, et al De novo mutations in FOXP1 in cases with intellectual disability, autism, and language impairment. American journal of human genetics. 2010;87(5):671–8. 10.1016/j.ajhg.2010.09.017 20950788 PMC2978954

[24] Horn D, Kapeller J, Rivera-Brugues N, Moog U, Lorenz-Depiereux B, Eck S, et al Identification of FOXP1 deletions in three unrelated patients with mental retardation and significant speech and language deficits. Human mutation. 2010;31(11):E1851–60. 10.1002/humu.21362 20848658 PMC3049153

[25] O'Roak BJ, Deriziotis P, Lee C, Vives L, Schwartz JJ, Girirajan S, et al Exome sequencing in sporadic autism spectrum disorders identifies severe de novo mutations. Nature genetics. 2011;43(6):585–9. 10.1038/ng.835 21572417 PMC3115696

[26] Talkowski ME, Rosenfeld JA, Blumenthal I, Pillalamarri V, Chiang C, Heilbut A, et al Sequencing chromosomal abnormalities reveals neurodevelopmental loci that confer risk across diagnostic boundaries. Cell. 2012;149(3):525–37. 10.1016/j.cell.2012.03.028 22521361 PMC3340505

[27] Le Fevre AK, Taylor S, Malek NH, Horn D, Carr CW, Abdul-Rahman OA, et al FOXP1 mutations cause intellectual disability and a recognizable phenotype. American journal of medical genetics Part A. 2013;161A(12):3166–75. 10.1002/ajmg.a.36174 24214399

[28] Gabut M, Samavarchi-Tehrani P, Wang X, Slobodeniuc V, O'Hanlon D, Sung HK, et al An alternative splicing switch regulates embryonic stem cell pluripotency and reprogramming. Cell. 2011;147(1):132–46. 10.1016/j.cell.2011.08.023 21924763

[29] Sarbassov DD, Guertin DA, Ali SM, Sabatini DM. Phosphorylation and regulation of Akt/PKB by the rictor-mTOR complex. Science. 2005;307(5712):1098–101. 15718470 10.1126/science.1106148

[30] Matsuda T, Cepko CL. Controlled expression of transgenes introduced by in vivo electroporation. Proceedings of the National Academy of Sciences of the United States of America. 2007;104(3):1027–32. 17209010 10.1073/pnas.0610155104 PMC1764220

[31] Ran FA, Hsu PD, Wright J, Agarwala V, Scott DA, Zhang F. Genome engineering using the CRISPR-Cas9 system. Nature protocols. 2013;8(11):2281–308. 10.1038/nprot.2013.143 24157548 PMC3969860

[32] Chen JG, Rasin MR, Kwan KY, Sestan N. Zfp312 is required for subcortical axonal projections and dendritic morphology of deep-layer pyramidal neurons of the cerebral cortex. Proceedings of the National Academy of Sciences of the United States of America. 2005;102(49):17792–7. 16314561 10.1073/pnas.0509032102 PMC1308928

[33] Saito T, Nakatsuji N. Efficient gene transfer into the embryonic mouse brain using in vivo electroporation. Developmental biology. 2001;240(1):237–46. 11784059 10.1006/dbio.2001.0439

[34] Nguyen L, Besson A, Heng JI, Schuurmans C, Teboul L, Parras C, et al p27kip1 independently promotes neuronal differentiation and migration in the cerebral cortex. Genes & development. 2006;20(11):1511–24. 16705040 10.1101/gad.377106 PMC1475763

[35] Cascio CJ. Somatosensory processing in neurodevelopmental disorders. Journal of neurodevelopmental disorders. 2010;2(2):62–9. 10.1007/s11689-010-9046-3 22127855 PMC3164038

[36] Baek ST, Kerjan G, Bielas SL, Lee JE, Fenstermaker AG, Novarino G, et al Off-target effect of doublecortin family shRNA on neuronal migration associated with endogenous microRNA dysregulation. Neuron. 2014;82(6):1255–62. 10.1016/j.neuron.2014.04.036 24945770 PMC4086250

[37] Straub C, Granger AJ, Saulnier JL, Sabatini BL. CRISPR/Cas9-mediated gene knock-down in post-mitotic neurons. PloS one. 2014;9(8):e105584 10.1371/journal.pone.0105584 25140704 PMC4139396

[38] Nagano T, Morikubo S, Sato M. Filamin A and FILIP (Filamin A-Interacting Protein) regulate cell polarity and motility in neocortical subventricular and intermediate zones during radial migration. The Journal of neuroscience: the official journal of the Society for Neuroscience. 2004;24(43):9648–57. 15509752 10.1523/JNEUROSCI.2363-04.2004 PMC6730158

[39] Tsai LH, Gleeson JG. Nucleokinesis in neuronal migration. Neuron. 2005;46(3):383–8. 15882636 10.1016/j.neuron.2005.04.013

[40] Bai J, Ramos RL, Ackman JB, Thomas AM, Lee RV, LoTurco JJ. RNAi reveals doublecortin is required for radial migration in rat neocortex. Nature neuroscience. 2003;6(12):1277–83. 14625554 10.1038/nn1153

[41] Guijarro P, Wang Y, Ying Y, Yao Y, Jieyi X, Yuan X. In vivo knockdown of cKit impairs neuronal migration and axonal extension in the cerebral cortex. Developmental neurobiology. 2013;73(12):871–87. 10.1002/dneu.22107 23843227

[42] Watanabe K, Takebayashi H, Bepari AK, Esumi S, Yanagawa Y, Tamamaki N. Dpy19l1, a multi-transmembrane protein, regulates the radial migration of glutamatergic neurons in the developing cerebral cortex. Development. 2011;138(22):4979–90. 10.1242/dev.068155 22028030 PMC3207862

[43] Zheng W, Geng AQ, Li PF, Wang Y, Yuan XB. Robo4 regulates the radial migration of newborn neurons in developing neocortex. Cerebral cortex. 2012;22(11):2587–601. 10.1093/cercor/bhr330 22123939 PMC4705339

[44] Zhang C, Mejia LA, Huang J, Valnegri P, Bennett EJ, Anckar J, et al The X-linked intellectual disability protein PHF6 associates with the PAF1 complex and regulates neuronal migration in the mammalian brain. Neuron. 2013;78(6):986–93. 10.1016/j.neuron.2013.04.021 23791194 PMC3694281

[45] Valiente M, Marin O. Neuronal migration mechanisms in development and disease. Current opinion in neurobiology. 2010;20(1):68–78. 10.1016/j.conb.2009.12.003 20053546

[46] Guerrini R, Dobyns WB. Malformations of cortical development: clinical features and genetic causes. The Lancet Neurology. 2014;13(7):710–26. 10.1016/S1474-4422(14)70040-7 24932993 PMC5548104

[47] Rubenstein JL. Annual Research Review: Development of the cerebral cortex: implications for neurodevelopmental disorders. Journal of child psychology and psychiatry, and allied disciplines. 2011;52(4):339–55. 10.1111/j.1469-7610.2010.02307.x 20735793 PMC3429600

[48] Guerrini R, Filippi T. Neuronal migration disorders, genetics, and epileptogenesis. Journal of child neurology. 2005;20(4):287–99. 15921228 10.1177/08830738050200040401

[49] Ackman JB, Aniksztejn L, Crepel V, Becq H, Pellegrino C, Cardoso C, et al Abnormal network activity in a targeted genetic model of human double cortex. The Journal of neuroscience: the official journal of the Society for Neuroscience. 2009;29(2):313–27. 19144832 10.1523/JNEUROSCI.4093-08.2009 PMC6664957

[50] Dierssen M, Ramakers GJ. Dendritic pathology in mental retardation: from molecular genetics to neurobiology. Genes, brain, and behavior. 2006;5 Suppl 2:48–60. 16681800 10.1111/j.1601-183X.2006.00224.x

[51] LaBerge D. Apical dendrite activity in cognition and consciousness. Consciousness and cognition. 2006;15(2):235–57. 16289990 10.1016/j.concog.2005.09.007

[52] Washington SD, Gordon EM, Brar J, Warburton S, Sawyer AT, Wolfe A, et al Dysmaturation of the default mode network in autism. Human brain mapping. 2014;35(4):1284–96. 10.1002/hbm.22252 23334984 PMC3651798

[53] Teramitsu I, Kudo LC, London SE, Geschwind DH, White SA. Parallel FoxP1 and FoxP2 expression in songbird and human brain predicts functional interaction. The Journal of neuroscience: the official journal of the Society for Neuroscience. 2004;24(13):3152–63. 15056695 10.1523/JNEUROSCI.5589-03.2004 PMC6730014

[54] Li S, Weidenfeld J, Morrisey EE. Transcriptional and DNA binding activity of the Foxp1/2/4 family is modulated by heterotypic and homotypic protein interactions. Molecular and cellular biology. 2004;24(2):809–22. 14701752 10.1128/MCB.24.2.809-822.2004 PMC343786

[55] Sin C, Li H, Crawford DA. Transcriptional Regulation by FOXP1, FOXP2, and FOXP4 Dimerization. Journal of molecular neuroscience: MN. 2014. 10.1007/s12031-014-0359-7 25027557

[56] Spiteri E, Konopka G, Coppola G, Bomar J, Oldham M, Ou J, et al Identification of the transcriptional targets of FOXP2, a gene linked to speech and language, in developing human brain. American journal of human genetics. 2007;81(6):1144–57. 17999357 10.1086/522237 PMC2276350

[57] Vernes SC, Spiteri E, Nicod J, Groszer M, Taylor JM, Davies KE, et al High-throughput analysis of promoter occupancy reveals direct neural targets of FOXP2, a gene mutated in speech and language disorders. American journal of human genetics. 2007;81(6):1232–50. 17999362 10.1086/522238 PMC2276341

[58] Vernes SC, Oliver PL, Spiteri E, Lockstone HE, Puliyadi R, Taylor JM, et al Foxp2 regulates gene networks implicated in neurite outgrowth in the developing brain. PLoS genetics. 2011;7(7):e1002145 10.1371/journal.pgen.1002145 21765815 PMC3131290

[59] Grundmann S, Lindmayer C, Hans FP, Hoefer I, Helbing T, Pasterkamp G, et al FoxP1 stimulates angiogenesis by repressing the inhibitory guidance protein semaphorin 5B in endothelial cells. PloS one. 2013;8(9):e70873 10.1371/journal.pone.0070873 24023716 PMC3759435

[60] Adams RH, Betz H, Puschel AW. A novel class of murine semaphorins with homology to thrombospondin is differentially expressed during early embryogenesis. Mechanisms of development. 1996;57(1):33–45. 8817451 10.1016/0925-4773(96)00525-4

[61] Lett RL, Wang W, O'Connor TP. Semaphorin 5B is a novel inhibitory cue for corticofugal axons. Cerebral cortex. 2009;19(6):1408–21. 10.1093/cercor/bhn179 18842660

